# Ovarian cancer-associated immune exhaustion involves SPP1+ T cell and NKT cell, symbolizing more malignant progression

**DOI:** 10.3389/fendo.2023.1168245

**Published:** 2023-04-18

**Authors:** Kunyu Wang, Hongyi Hou, Yanan Zhang, Miao Ao, Haixia Luo, Bin Li

**Affiliations:** Department of Gynecology Oncology, National Cancer Center/National Clinical Research Center for Cancer/Cancer Hospital, Chinese Academy of Medical Sciences and Peking Union Medical College, Beijing, China

**Keywords:** ovarian cancer, SPP1+ T cell, T cell exhaustion, single cell RNA sequencing, immune environment

## Abstract

**Background:**

Ovarian cancer (OC) is highly heterogeneous and has a poor prognosis. A better understanding of OC biology could provide more effective therapeutic paradigms for different OC subtypes.

**Methods:**

To reveal the heterogeneity of T cell-associated subclusters in OC, we performed an in-depth analysis of single-cell transcriptional profiles and clinical information of patients with OC. Then, the above analysis results were verified by qPCR and flow cytometry examine.

**Results:**

After screening by threshold, a total of 85,699 cells in 16 ovarian cancer tissue samples were clustered into 25 major cell groups. By performing further clustering of T cell-associated clusters, we annotated a total of 14 T cell subclusters. Then, four distinct single-cell landscapes of exhausted T (Tex) cells were screened, and SPP1 + Tex significantly correlated with NKT cell strength. A large amount of RNA sequencing expression data combining the CIBERSORTx tool were labeled with cell types from our single-cell data. Calculating the relative abundance of cell types revealed that a greater proportion of SPP1 + Tex cells was associated with poor prognosis in a cohort of 371 patients with OC. In addition, we showed that the poor prognosis of patients in the high SPP1 + Tex expression group might be related to the suppression of immune checkpoints. Finally, we verified *in vitro* that SPP1 expression was significantly higher in ovarian cancer cells than in normal ovarian cells. By flow cytometry, knockdown of SPP1 in ovarian cancer cells could promote tumorigenic apoptosis.

**Conclusion:**

This is the first study to provide a more comprehensive understanding of the heterogeneity and clinical significance of Tex cells in OC, which will contribute to the development of more precise and effective therapies.

## 1 Introduction

Ovarian cancer (OC) is one of the deadliest and most aggressive tumors in women, and its incidence has increased in recent years (1). Because the early specific signs and symptoms of OC are not obvious and develop rapidly, the vast majority of patients with OC are not diagnosed until the late stage (2). Patients with OC often have a poor prognosis, presumably because their heterogeneity limits reproducible prognostic classification (3). At present, surgery, chemotherapy and radiotherapy are the most common modalities used in the treatment of OC. However, the side effects of treatment in these patients are more severe and there is a serious decrease in the quality of life of the patients (4). Extensive heterogeneity in OC cells is a critical mechanism for overall survival and cancer progression (5). Therefore, it is of great significance to explore specific markers for the early diagnosis of OC to improve treatment and patient outcomes.

Emerging single-cell technologies provide powerful tools to explore heterogeneity and thereby aid in problem solving (6, 7). This technology has been increasingly used in the field of cancer and provides new mechanisms for understanding carcinogenesis and revealing therapeutic strategies (8–12). However, only a few studies have investigated OC at the single-cell level. A recent single-cell RNA sequencing (scRNA-seq) study investigated tumor heterogeneity at cellular resolution using OC samples (13). Another study examined how fallopian tube epithelial cell sources could accurately predict cancer behavior (14). These studies provide new insights into the carcinogenesis of OC, and their findings enhance our understanding of OC.

Tumors are characterized by significant heterogeneity that can lead to differential responses to the same therapy (15). Until now, there have been efforts to explore the heterogeneous characteristics of tumors. However, our understanding of tumor heterogeneity is still limited to tumor cells due to the limitations of technology. Recent studies have demonstrated that tumor-infiltrating immune and stromal cells exhibit heterogeneity (16). In addition, increasing evidence suggests that the tumor microenvironment (TME) plays an important role in targeting agents (17). Previous studies have highlighted CD8+ failure, immune checkpoints, and tumor-associated macrophages as key therapeutic targets (18, 19). These data enhance our understanding of TME heterogeneity.

To reveal the heterogeneity of T cell-associated subclusters in OC, we performed an in-depth analysis of single-cell transcriptional profiles and clinical information of patients with OC. We then explored the immune landscape of four different Tex and could clearly see a significant correlation between SPP1 + Tex and NKT cells. A large amount of RNA-seq expression data combining the CIBERSORTx tool and TCGA were labeled with cell types from our single-cell data. In calculating the abundance of immune cells for each patient, we found that the abundance of SPP1 + Tex cells was associated with poor prognosis. In addition, we showed that the poor prognosis of patients in the high SPP1 + Tex expression group might be related to the suppression of immune checkpoints. Finally, we performed *in vitro* experiments for validation. The expression level of SPP1 in ovarian cancer cells was significantly higher than that in normal ovarian cells, and the ability to promote apoptosis after knocking down SPP1 in ovarian cancer cells could be seen by flow cytometry. This is the first study to provide a more comprehensive understanding of the heterogeneity and clinical significance of Tex cells in OC, which will contribute to the development of more precise and effective therapies.

## 2 Materials and methods

### 2.1 Data collection

Three single-cell datasets (E-MTAB-8107, GSE154600, and GSE130000) were obtained from the Gene Expression Omnibus (http://www.ncbi.nlm.nih.gov/geo/) containing a total of 16 samples from patients with OC. RNA-seq data and accompanying clinical information from 371 OC samples were downloaded from the TCGA cohort for further correlation analysis (http://cancergenome.nih.gov/). This study used a publicly available dataset that received ethical approval from the original study.

### 2.2 Data filtering and correction

We used the “Seurat” and “SingleR” software packages for scRNA-seq data analysis. We filtered cells with unique feature counts > 2500 or < 200 and cells with mitochondrial counts > 5%. Then, the feature-expression measurements for each cell were normalized to the total expression using the default parameters of the Seurat “NormalizeData” function. Subsequently, all cell data were transferred to a combined Seurat object using the Harmony package. Variable genes were then scaled, and the principal component (PC) was analyzed. Using the “RunUMAP” (min. dist = 0.2, n. neighbors = 20) and “FindClusters” (resolution = 0.5) functions, significant PCs were selected for Umap and cluster analyses.

### 2.3 Cell annotation

To identify cell types, we performed two annotation modalities. Automated annotation (used for the first clustering to select T cell-related subsets): SingleR is an automated annotation method for scRNAseq data (20). Given a sample reference dataset (single cell or batch size) with known labels, it marks new units in the test dataset based on their similarity to the reference. Thus, for reference datasets, the burden of manually interpreting clusters and defining marker genes only needed to be done once, whereas biological knowledge could be spread to new datasets in an automated manner.

Manual annotation (used to cluster T cell-related subsets for the second time): We checked whether the well-studied marker genes were the top differentially expressed genes (DEGs) and annotated the most likely identity for each cell cluster. The remaining cell types were identified by manually searching the cell marker database (http://biocc.hrbmu.edu.cn/CellMarker/). The R package “estimate” was used for estimate analysis to classify and score cells as a whole: estimate score, immune score, and stromal score.

### 2.4 GSEA pathway and cell-to-cell communication analyses

We performed GSEA pathway and cell-to-cell communication analyses to explore the association between T cell-associated subsets. R package “ABGSEase” was used to perform biological pathway enrichment between the two groups, and the reference gene set was Hallmark, GO, and KEGG. Minimum gene set size minGSSize = 50, maximum gene set size maxGSSize = 100, and P-value truncated at P-value cutoff = 0.05 were set for the analysis. Cell–cell communication analysis uses the R package “CellChat”, and the pathway selects the secreted signaling pathway. The reference human ligand–receptor database was CellChatDB. We examined the interactions between different cell types and filtering pathways with cell numbers less than 10.

### 2.5 Unsupervised consensus cluster analysis

Robust Tex cell infiltration-associated clusters can be found in TCGA cohort patients by consensus clustering techniques based on partitioning and expression of Tex cells in 4 with the help of the R package “ConsensuClusterPlus”. The cumulative distribution function and consensus heat map were used to determine the optimal K-value. The method was repeated 1000 times to ensure the stability of the layering process.

### 2.6 Prognostic analysis

For the selected cells, univariate cox regression analysis was first performed to select prognostically relevant Tex cells (P < 0.05). Kaplan–Meier curves were used to assess the differences in survival between the high and low groups of such cells.

### 2.7 Immune infiltrate analysis

Immune infiltration analysis was performed using the CIBERSORTx algorithm (21), which quantifies the absolute content of 22 immune cells based on the patient’s transcriptional profile information, as well as the absolute content of Tex cell infiltrates in 4 derived from a reference dataset of our own single-cell data.

### 2.8 DEG analysis

The main purpose of this analysis was to identify DEGs between the SPP1 + Tex high and low groups. DEG analysis was performed using the “limma” package in R software with thresholds set at log FoldChange ≥ 1 and adj PVal Filter (adj P) < 0.05. Subsequently, GSEA was performed for the SPP1 + Tex high and low groups to explore the significance of their biological functions. Finally, we analyzed the expression of immune checkpoints in the high and low SPP1 + Tex groups.

### 2.9 Cell culture

Human normal ovarian cells IOSE80 and ovarian cancer cells A2780 were purchased from American Type Culture Collection (ATCC, Rockville, MD, USA). Cells were cultured in RPMI-1640 medium containing 10% Fetal Bovine Serum (FBS) at 37° C and 5% CO2.

### 2.10 Quantitative real-time PCR (qRT-PCR)

Cells were treated with TRIzol reagent (Takara, Japan). We then extracted all RNA and reverse-transcribed it into cDNA. qRT-PCR was used to analyze the relative expression of SPP1, and data were normalized to GAPDH. Reverse transcription system: 500ng RNA, 2ul RT Master Mix, add RNase-free water to fix the volume to 10μl. qPCR system: 10μl 2xTB Green, 8ul ddH2O, 1μl cDNA, 1μl primer (22). The primer sequences are as follows: SPP1-F::5’-AGA CCC TGA CAT CCA GTA CCT G-3’, SPP1-R: 5’-GTG GGT TTC AGC TAC CTG GT-3’. GAPDH-F: 5’-GGAGCGAGATCCCTCCAAAAT-3’, GAPDH-R: 5’-GGCTGTTGTCATACTTCTCATGG-3’.

### 2.11 Apoptosis analysis

We analyzed cell apoptosis using flow cytometry after pre-cooling PBS washing and digestion with trypsin digestion solution containing no EDTA (Solarbio, Shanghai, China). Cells were harvested after centrifugation at 1000 rpm for 5 minutes, followed by 7-AAD (BD Biosciences, number 559, 925, USA) staining and annexin-APC (BD Biosciences, number 561, 012, USA) staining for 15 minutes.

### 2.12 Statistical analysis

The student’s t-test was used for normally distributed continuous variables. The Mann–Whitney U test was used for continuous variables that were not normally distributed. Correlations between continuous variables were evaluated using Pearson’s correlation analysis. Statistical significance was set at P < 0.05. R software version 4.1.0 (http://www.R-project.org) was used for data analysis and figure generation.

## 3 Results

**Figure 1:**
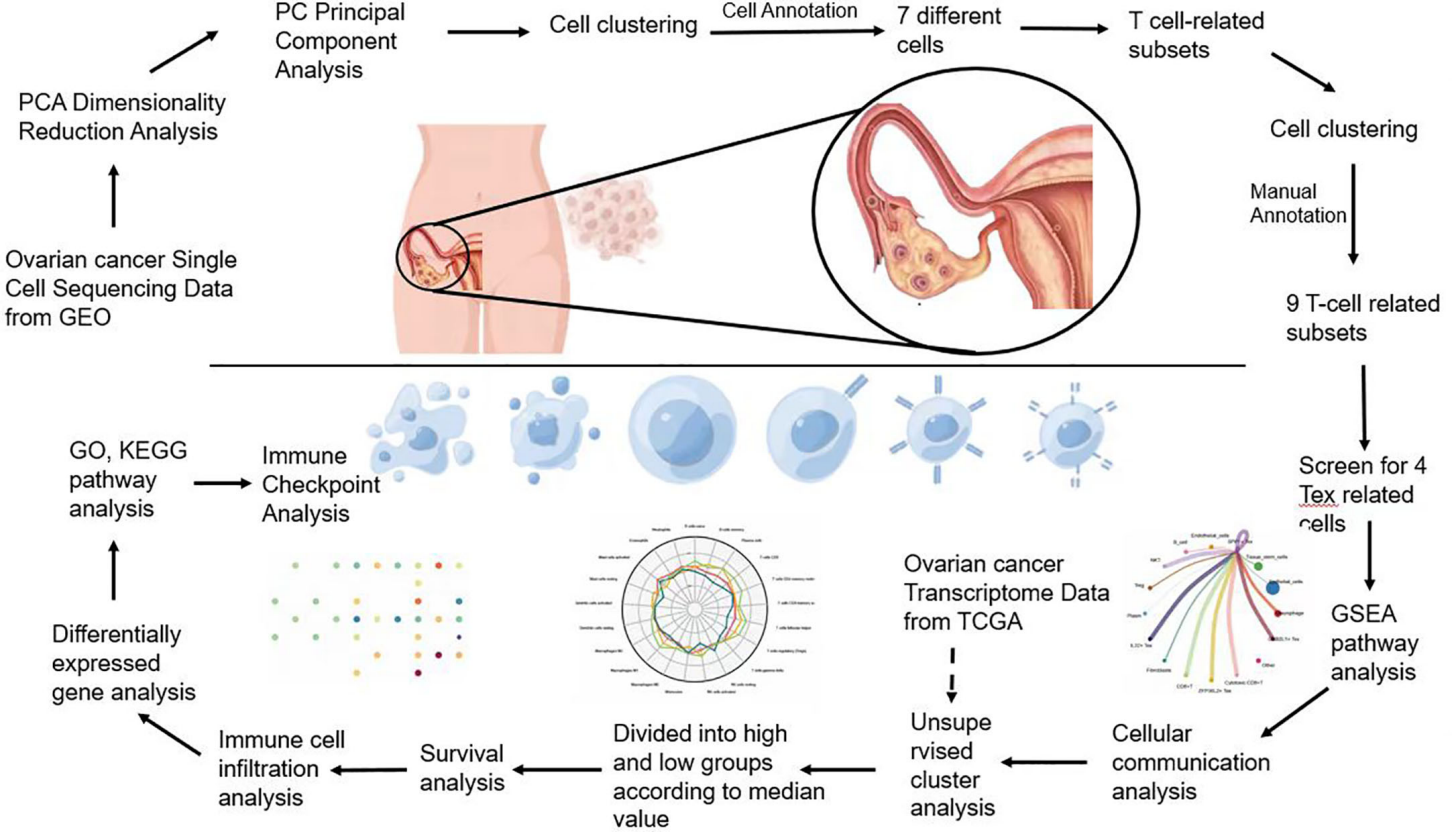
Flowchart.

Flowchart (**Figure 1**).

### 3.1 Cell clustering of OC landscapes

**Figure 2:**
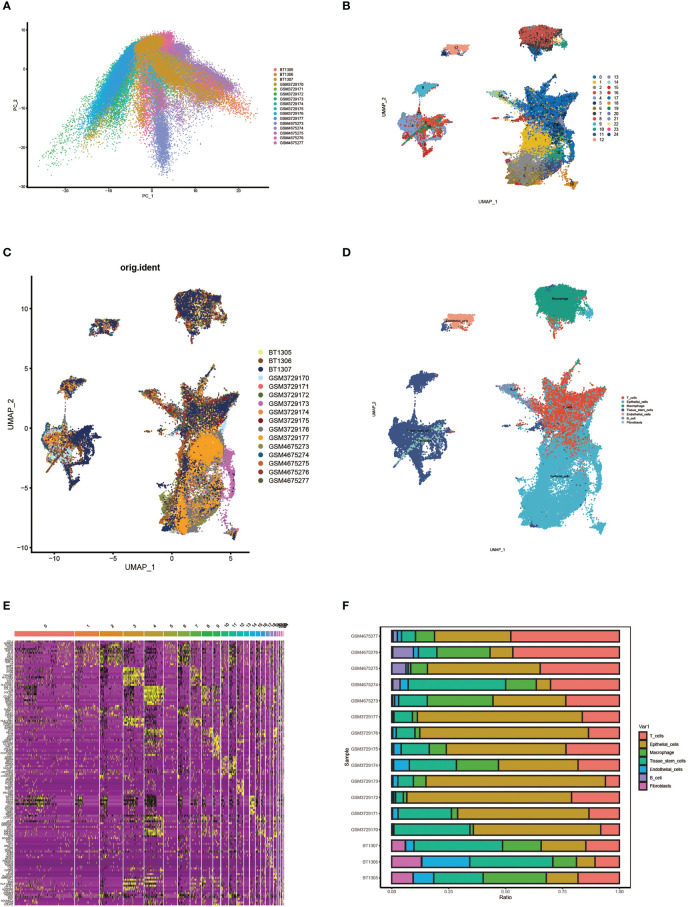
Single cell cluster analysis in patients with ovarian cancer. **(A)** Single cell data were subjected to PCA dimensionality reduction analysis and the first 50 PC principal components were selected for subsequent analysis. **(B)** Umap plots show 25 clusters of cells generated by clustering after dimension reduction. **(C)** Umap plot showing cell distribution for different samples. **(D)** Umap plots show the distribution of cells in each cluster after annotation. **(E)** Heat map of signature gene expression in different groups. **(F)** Histograms show the proportion of infiltrates per cluster of cells in each sample.

First, we performed principal component analysis (PCA) on 16 samples to reduce dimensionality and selected the first 50 PCs for subsequent analysis (**Figure 2A**). Following data processing and screening, we obtained gene expression profiles for 85,699 cells from 16 OC samples and identified 25 cell clusters using Seurat (**Figure 2B**). Cell distributions are visualized by Umap plots for different samples (**Figure 2C**). Cells in clusters 0 and 20 were classified as T cells by defining the annotation of cell identity in each cluster by cross-referencing the DEGs in each cluster to canonical marker genes (**Figure 2D**). The heatmap visualizes the expression of genes in each cluster of cells, with yellow highlighted sections representing genes highly expressed in this cluster (**Figure 2E**). The expression levels of some signature genes in this cluster were visualized using violin plots (**Supplementary Figure 1**). In addition, we showed the infiltrative content of seven clusters of cells in each sample by histogram and found that epithelial cells accounted for the highest proportion in most samples (**Figure 2F**).

### 3.2 Cellular clustering of T cell subsets in OC

We calculated three scores for the three classes of cells using the package “estimate”. Immune cells had the highest immune score. tumor cells had the highest tumor purity score, and other cells had the highest stromal score (**Supplementary Figure 2**). This score also demonstrates the accuracy of the grouping.

**Figure 3:**
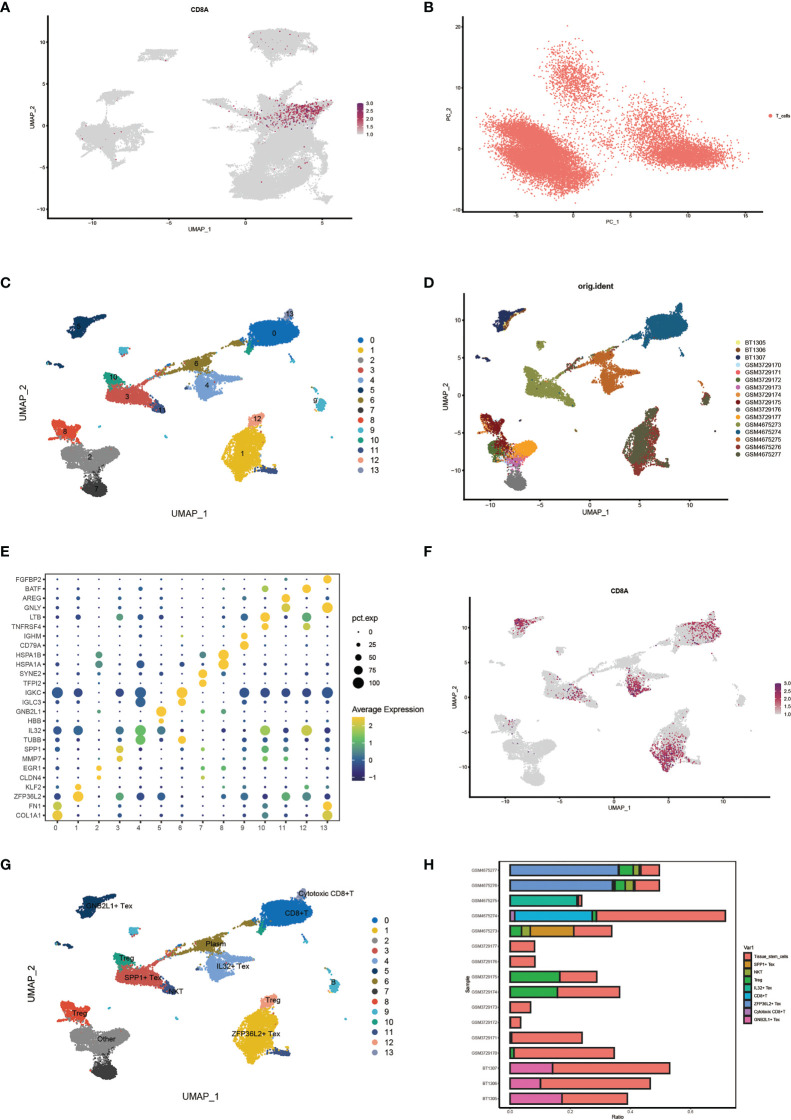
Identification of characteristic cell clusters. **(A)** View CD8 + T cell location by distribution of cell signature genes. **(B)** PCA Dimensionality Reduction Analysis of T Cell Clusters. **(C)** Umap shows 14 clusters of cells after dimension reduction. **(D)** Umap Plot of cell distribution by sample. **(E)** Bubble plots showing signature genes for each T cell subcluster. **(F)** Umap plot showing distribution of CD8marker. **(G)** Umap plots show results after annotation of T-cell clusters. **(H)** Histogram plot showing cell proportions for each sample.

First, we identified CD8+ T cell locations by determining the distribution of cell signature genes (**Figure 3A**) and proceeded with PCA dimensionality reduction of the T cell clusters (**Figure 3B**). Subsequently, Umap dimensionality reduction was performed to obtain 14 clusters of cells, and the cell distribution of different samples is shown (**Figures 3C, D**). Through bubble plots, we can visually observe the signature genes of each T cell subcluster (**Figure 3E**). The Umap plot shows the distribution of CD8A markers (**Figure 3F**). By determining the distribution of the Tex cell marker, clusters 5, 3, 4, and 1 were identified as CD8+ Tex cells based on this distribution (**Supplementary Figure 3A**). Clusters 8, 10, and 12 were identified as Treg cells based on the distribution of the two Treg cell markers (**Supplementary Figure 3B**). Finally, the results for T cell subsets were determined by manual annotation, with 14 cell clusters annotated as a total of nine Tex-related cell subsets (**Figure 3G**). Using the Umap plot, we determined the distribution of highly expressed genes in Tex cells (**Supplementary Figure 3C**). In addition, we determined the proportion of Tex cells in the samples using a histogram plot (**Figure 3H**). We found that the content of T cells in different samples was significantly different.

### 3.3 Pathway analysis of four Tex cells

**Figure 4:**
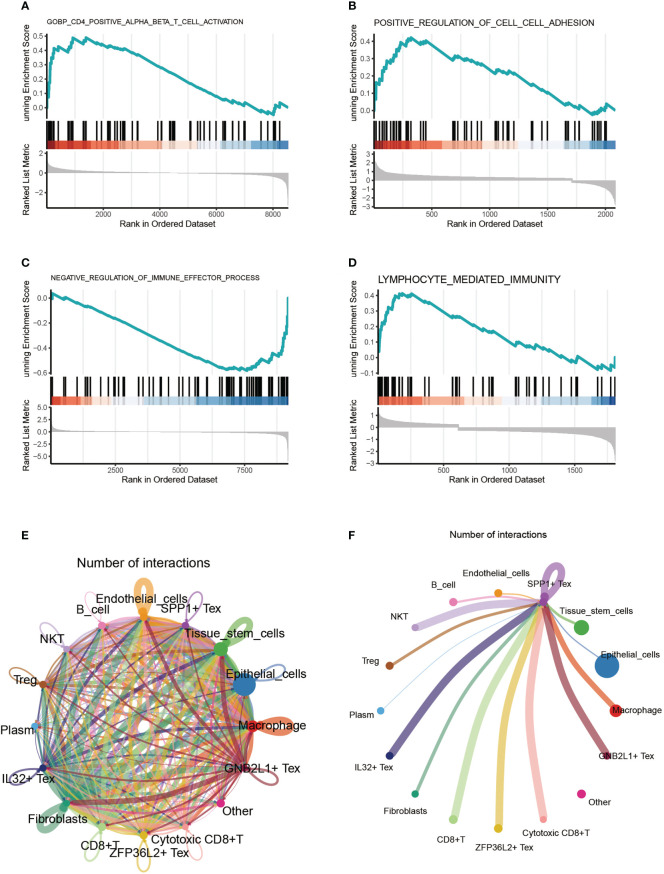
Cell-enriched pathway analysis. **(A-D)** GSEA analysis comparing enriched pathways in four Tex cells. **(E)** Shown the pathway analysis between cells. **(F)** Signaling pathway of SPP1 + Tex to other cells.

By comparing the enriched pathways in four Tex cells using GSEA analysis, we found that SPP1 + cluster CD4+ αβ T cells were functionally active (**Figure 4A**). Comparing the SPP1 + Tex and ZFP36S2 + Tex cluster cells, we found that the positive regulation of cell adhesion was significantly activated (**Figure 4B**). GNB2L1 + Tex cluster cells showed activation of negative regulation of immune effector processes (**Figure 4C**). IL32 + Tex cluster cells showed significant activation of the lymphocyte-mediated immune function (**Figure 4D**). Cell communication analysis revealed a close connection between these cells (**Figure 4E**). In addition, we found that the signal emitted by SPP1 + Tex was very strong in NKT cells, in addition to a significant link with IL32 + Tex, GNB2L1 + Tex, and other cells (**Figure 4F**).

### 3.4 Identification of the role of Tex cell-related pathways

**Figure 5:**
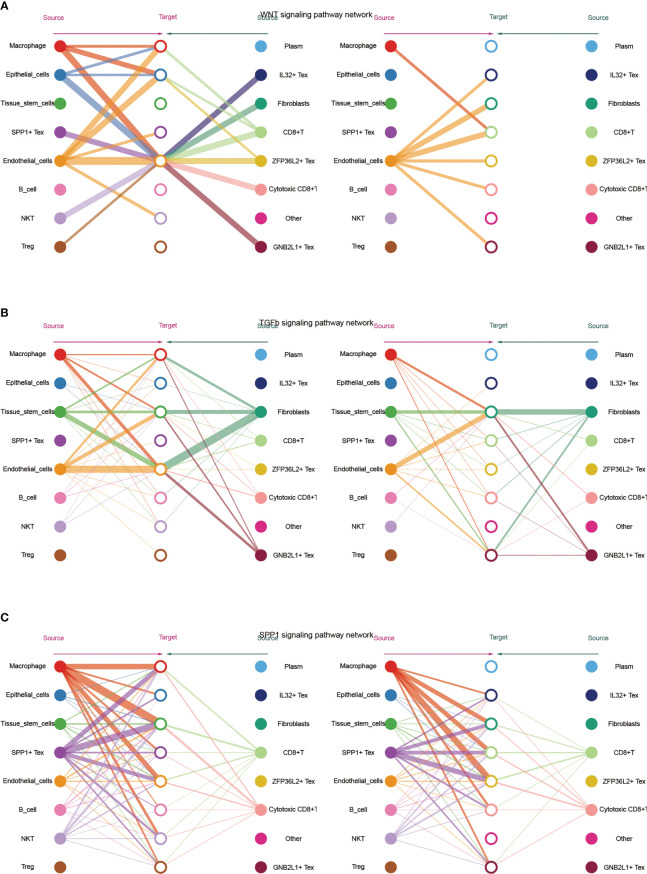
Network diagram showing **(A)** WNT, **(B)** TGF -β and **(C)** SPP1 related signaling pathways, respectively. The role of TGF-β-related pathways among all cells was shown by bubble plots, in which TGF-β 1 (TGF-β R1 + TGF-β R2) was generally more active among various types of cell communication (**Figure 6A**). In addition, analysis of the effect of related pathways between Tex cells showed that the effect of BMP4- and GDF5-related pathways differed between Tex cells, in which GNB2L1 + Tex actively interacted with stem cells, while SPP1 + Tex cells communicated mainly with stem cells through BMP4 (BMPR1B + ACVR2A) and GDF5 (BMPR1B + ACVR2A) (**Figure 6B**).

Subsequently, we visualized the cellular role of the sub-pathways of secretory cell communication. Cell communication diagram shows the signaling pathway networks of WNT, TGF-β, and SPP1. The results showed that endothelial cells expressed the WNT signaling pathway significantly, and the WNT-based pathway macrophage Tex cells had a strong effect on endothelial cells (**Figure 5A**). In addition, fibroblasts were more potent based on the TGF-β pathway (**Figure 5B**). Based on the fact that SPP1 + Tex is highly active in the SPP1 signaling pathway, it was demonstrated that the main effect of SPP1 + Tex is from its marker SPP1 and that it may interact with fibroblasts (**Figure 5C**).

**Figure 6:**
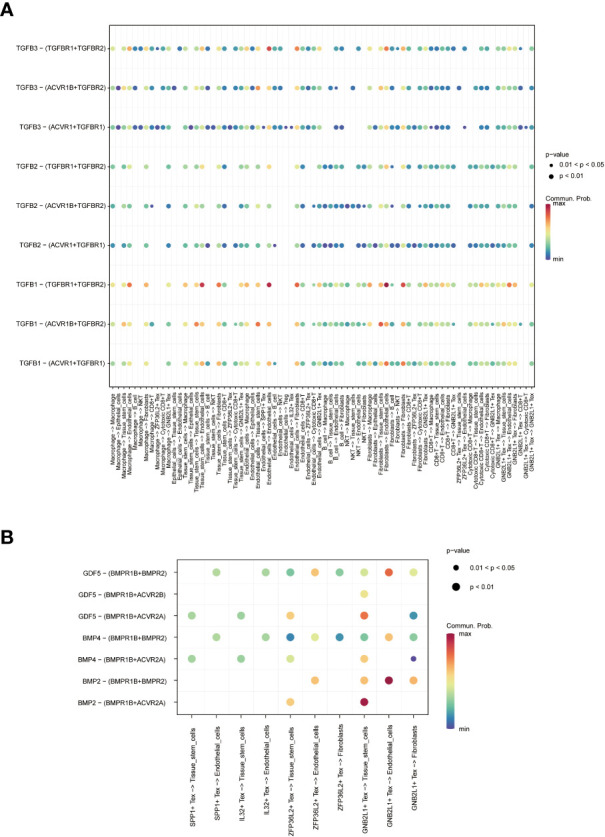
Analysis of cell-related pathways. **(A)** Bubble plots demonstrate the role of TGF-β related pathways across all cells. **(B)** Bubble plots demonstrate the role of Tex cell-associated pathways.

### 3.5 Identification of the prognostic role of Tex cells

**Figure 7:**
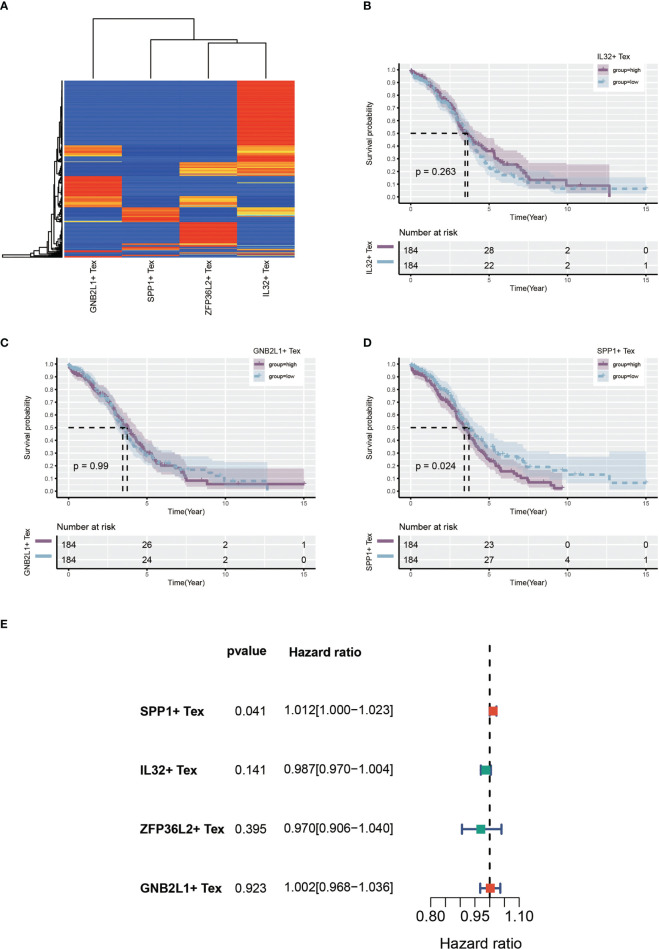
Identify Tex-related cellular features. **(A)** Heat map showing expression of four Tex-related cells in the TCGA cohort. **(B-D)** Survival curves showing survival differences between the three Tex cells in the high and low groups divided according to the median. **(E)** Forest plot showing the results of univariate cox regression.

Using single-cell data as reference data, Tex-related cell content was calculated from the OC dataset in TCGA using the CIBERSORTx algorithm. A heat map showing the expression of four cells in the TCGA cohort was created (**Figure 7A**). Patients with OC were divided into two groups according to the median expression level. Many samples had expression values of 0 in ZFP36L2 + Tex cells, which may have affected the analytical results. hence, we did not perform subsequent analysis on them. Survival curves showed differences in survival between the high and low groups of the three Tex cells, with IL32 + Tex and GNB2L1 + Tex cells not being associated with survival (**Figures 7B, C**), whereas SPP1 + Tex cells showed a correlation with survival, and the high group had a poor prognosis (**Figure 7D**). In addition, univariate cox regression demonstrated that SPP1 + Tex cells are an unfavorable prognostic factor for OC (**Figure 7E**).

### 3.6 Identification of components of immune cell infiltration of Tex cells and their correlation

**Figure 8:**
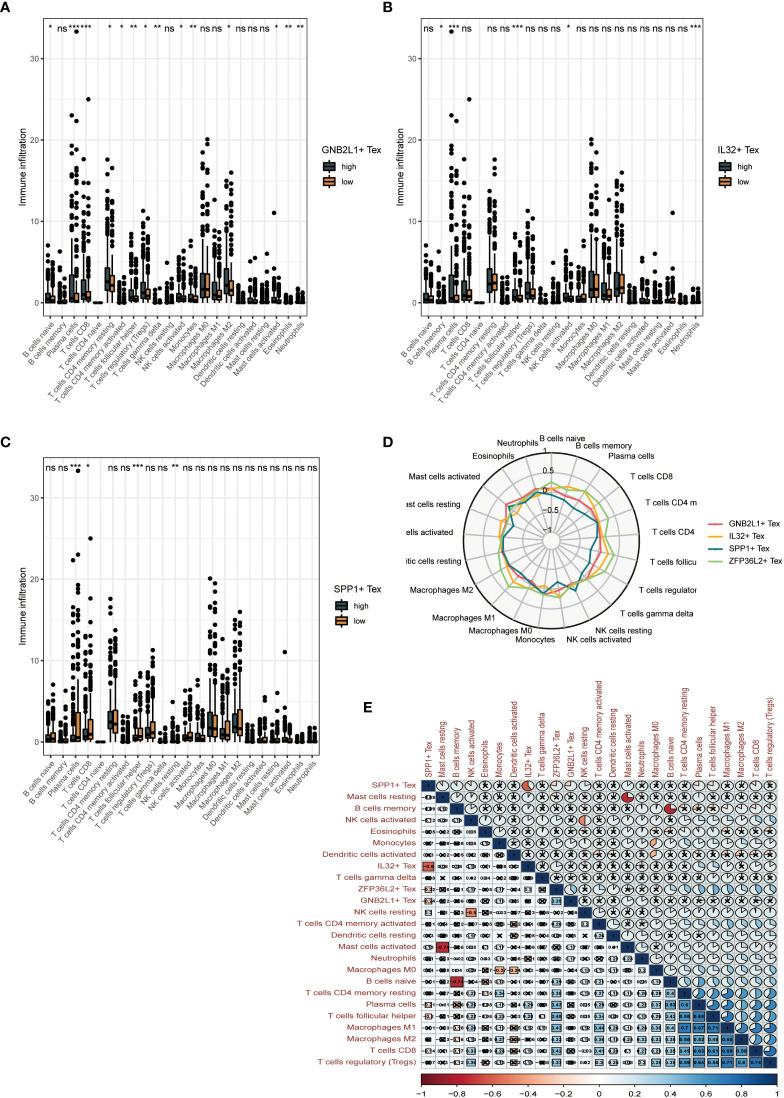
Clinical value of Tex cell clusters identified by our scRNA-seq analysis in patients from the TCGA OC cohort. **(A-C)** Difference in overall cellular infiltration between high and low groups according to median values for the 3 Tex cells. **(D)** Radar plots show the correlation of 4 cells with other immune cells. **(E)** Heat map showing correlation between all cells. * means <0.05,** means <0.01,*** means <0.001. ns means >0.05.

By performing immune cell infiltration analysis between the high and low groups of three Tex cells, we found significant differences in immune cell composition between the high and low groups. The results showed a significant difference between plasma cells and CD8 T cells in the high and low GNB2L1 + Tex cell groups (**Figure 8A**). There were significant differences in plasma cells, follicular helper T cells, and neutrophils between the high and low IL32 + Tex groups (**Figure 8B**).

Plasma cells, CD8 T cells, follicular helper T cells, and NK cells were significantly different between the high and low SPP1 + Tex cell groups (**Figure 8C**). The proportion of helper infiltration of plasma cells, CD8 T cells, and T cell follicles in the low-expression group was significantly higher than that in the high-expression group, whereas the proportion of NK cell infiltration in the high-expression group was higher than that in the low-expression group. This result suggests that the difference in survival between the high and low SPP1 + Tex groups may be due to improved immune control.

Radar plots showed the correlation between the four Tex cells and other immune cells (**Figure 8D**). A significant negative correlation between SPP1 + Tex and IL32 + Tex cells, and a negative correlation was found between SPP1 + Tex and plasma cells, T cell follicular helper using correlation heat maps (**Figure 8E**). In addition, the correlation analysis of the 4 Tex cells also showed the strongest correlation between SPP1 + Tex and IL32 + Tex cells (**Supplementary Figure 4**).

### 3.7 Identification of differences between high and low SPP1 + Tex cell groups

**Figure 9:**
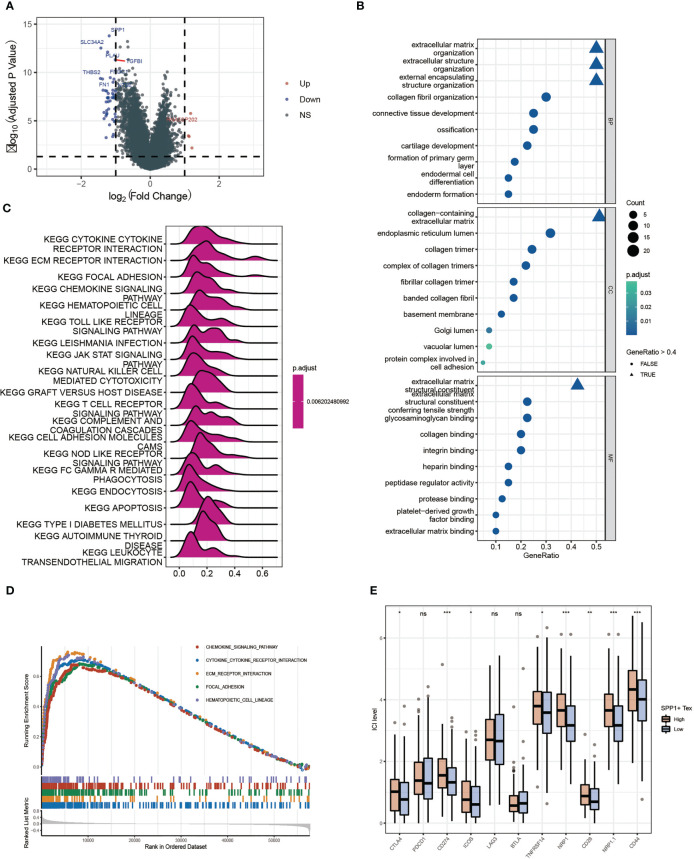
Differential enrichment analysis between high and low SPP1 + Tex groups. **(A)** Volcano plot of differentially expressed genes analysis between high and low groups. **(B)** GO enrichment analysis bubble plot. **(C)** Ridge plots for KEGG enrichment analysis. **(D)** Pathway plots for GSEA enrichment analysis. **(E)** Box plots show results of immune checkpoint analysis between SPP1 + Tex high and low groups. * means <0.05,** means <0.01,*** means <0.001. ns means >0.05.

By performing differential gene expression analysis between the SPP1 + Tex high and low groups, we drew a volcano plot for visualization (**Figure 9A**). GO analysis revealed that these DEGs were enriched in terms of extracellular matrix. therefore, SPP1 + Tex may be associated with extracellular matrix remodeling (**Figure 9B**). GSEA analysis, based on KEGG data, showed that DEGs were significantly enriched in chemokine signaling pathways, cytokine receptor interactions, ECM receptor interactions, and local adhesion signaling pathways (**Figures 9C, D**). In addition, the expression of immune checkpoints in the SPP1 + Tex high and low groups was analyzed, and the results showed significant differences in CD274, NRP1, NRP1.1, CD28, and CD44 between the high and low groups (**Figure 9E**). Interestingly, the number of patients in the high SPP1 + Tex expression group was larger than that in the low SPP1 + Tex expression group among these immune checkpoint inhibitors, corresponding to the worse outcome in the high-expression group.

### 3.8 *In vitro* validation

**Figure 10:**
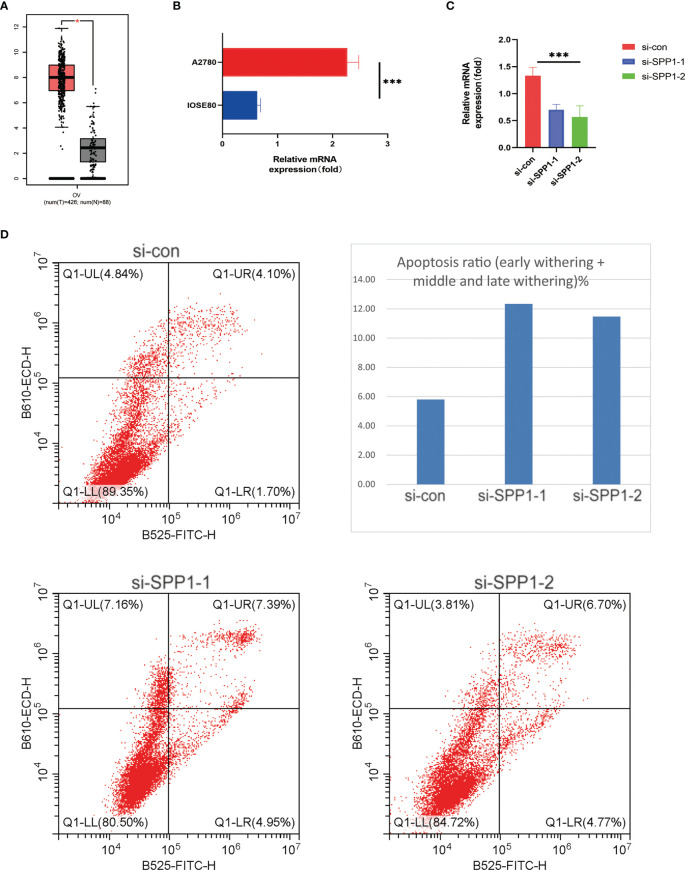
*In vitro* experiments validated SPP1 as a potential target. **(A)** Boxplot showing differential expression of SPP1 gene between ovarian cancer patients and normal patients. **(B)** Histogram showing expression levels of SPP1 gene in normal ovarian cells and ovarian cancer cells. **(C)** Histogram showing knockdown of SPP1 gene expression levels in ovarian cancer cells. **(D)** Flow cytometry scatter plot showed that SPP1 gene could affect apoptosis of ovarian cancer cells. *** means <0.001.

With the previous results, it can be seen that only SPP1 + Tex has prognostic value in KM analysis and COX analysis. Therefore, we mainly chose SPP1 as the subject of further study in our subsequent study. To validate the validity of our model and identify a potential biomarker, we performed *in vitro* experimental validation from selection of SPP1. It can be found by boxplots that SPP1 has a very high expression level in ovarian cancer patients (**Figure 10A**). The SPP1 gene was expressed at a significantly higher level in ovarian cancer cells A2780 than in normal ovarian cells IOSE80, which also demonstrated the accuracy of our experiment (**Figure 10B**). In addition, we knocked down the expression level of SPP1 gene in A2780 cells and quantified it again to verify our knockdown efficiency (**Figure 10C**). By flow cytometry, we analyzed the function of SPP1 in ovarian cancer. The results showed that knockdown of SPP1 significantly promoted apoptosis in ovarian cancer cells (**Figure 10D**). Therefore, SPP1 may be a potential therapeutic target for ovarian cancer.

## 4 Discussion

The past decade has witnessed a shift in the paradigm of cancer therapy with the advent of approaches to target or manipulate the immune system (“immunotherapy”) (23–25). Cancer cells are often immunogenic while in the organism, but the immune system is often unable to clear it. This is because cancer cells have mechanisms that prevent recognition by the immune system, including central tolerance, ignorance or failure to be activated in the periphery, extrinsic regulation of T cells (e.g., regulatory T cells, myeloid-derived suppressor cells, and suppressor cytokines), and intrinsic dysfunction of T cells, resulting in inappropriate or excessive antigenic stimulation (anergy and depletion) (26–28). Antibodies targeting inhibitory pathways, including CTLA-4 and PD-1, have paved the way for a new generation of cancer therapeutics (29–31).

T cell depletion is characterized by the overexpression of multiple inhibitory receptors, including PD-1 (CD279), cytotoxic T-lymphocyte antigen-4 (CTLA-4, CD152), lymphocyte activation gene 3 (Lag-3), T cell immunoglobulin domain and mucin domain 3 (Tim-3), CD244/2B4, CD160, T cell immune receptor-containing Ig and ITIM domains (TIGIT), and other receptors (32). Blocking the PD-1 pathway partially reverses failure and leads to reduced viral or tumor burden, which is a breakthrough (33, 34). These data suggest that Tex is not an ultimate dysfunction but can be revitalized and is important for the treatment of diseases, including cancer.

To reveal the heterogeneity of T cell-associated subclusters in OC, we performed an in-depth analysis of single-cell transcriptional profiles and clinical information of patients with OC. By performing further clustering of T cell-associated clusters, we annotated a total of 14 T cell subclusters. We then explored the immune landscape of four different Tex and could clearly see a significant correlation between SPP1 + Tex and NKT cells. A large amount of RNA-seq expression data combined with the CIBERSORTx tool and TCGA were labeled with cell types from our single-cell data. In all OC patients, we found that the higher the abundance of SPP1 + Tex cells, the worse prognosis of the patients.

We found a greater association between SPP1+ Tex and NKT cells by cell communication analysis. NKT cells are T cells with T-cell receptors that primarily recognize lipid antigens presented by CD1d. In cancer, NKT cells tend to play different roles, and type I NKT cells, which activate NK and CD8+ T cells by producing interferon-γ, are mostly protective (35). In contrast, type II NKT cells, characterized by a more diverse T cell receptor recognizing CD1d-presented lipids, predominantly suppress tumor immunity (36). Moreover, type I and II NKT cells counter-regulate each other and form a novel immunomodulatory axis (35). Thus, manipulating this balance along the NKT regulatory axis may be critical for cancer immunotherapy.

In addition, we found that SPP1 + Tex significantly enhanced the regulation of cell adhesion compared to other Tex cells. Unlike most other tumor types that metastasize *via* the vasculature, OC metastasizes predominantly *via* the transcavitary route within the peritoneal cavity (37). In the peritoneal cavity, tumor-mesothelial adhesion is an important step in cancer dissemination (38). therefore, we reasoned that cell adhesion pathways could be potential pathways to inhibit OC.

Immunocyte infiltration analysis showed that the proportion of plasma cell, CD8 T cell, and follicular helper T cell infiltration in the low-expression group was significantly higher than that in the high-expression group. Plasma cell infiltration in OC has a significant impact on tumor progression and prognosis (39). Follicular helper T cells are specialized providers of T cells that contribute to B cells and the formation of germinal center responses, and numerous studies have demonstrated their important role in various malignancies (40, 41). Immune checkpoint inhibitor analysis revealed that the levels of immune checkpoint inhibitors were significantly higher in the high SPP1 + Tex expression group than in the low SPP1 + Tex expression group. This corresponds to the outcome of poor prognosis in the high-expression group. Additionally, patients in the high SPP1 + Tex group may benefit more from anti-immune checkpoint inhibitors. In addition, we found that only SPP1 + Tex had better prognostic efficacy among the four previously studied Tex. Therefore, we selected SPP1 for further study in ovarian cancer. Finally, we verified *in vitro* that SPP1 expression was significantly higher in ovarian cancer cells than in normal ovarian cells. By flow cytometry, knockdown of SPP1 in ovarian cancer cells could promote tumorigenic apoptosis. SPP1 may be a potential therapeutic target for ovarian cancer.

Also, we must acknowledge the potential limitations of our analysis. First of all, our study is based on the analysis of public databases. Therefore, further multicenter, large sample, prospective studies that may follow are needed. Secondly, the screened gene SPP1 was only partially phenotypically experimented, and further exploration about the molecular mechanism needs to be followed up.

In addition, the cell type-specific marker expression patterns described in this study may contribute to a better understanding of the heterogeneity and biological characteristics of OC. The present work revealed markers for cells of different Tex subsets that may be better in diagnostics or other biological experiments. In conclusion, our study provides new insights into the heterogeneity of OC and may contribute to the development of new and efficient therapies for OC.

## 5 Conclusions

This is the first study to provide a more comprehensive understanding of the heterogeneity and clinical significance of Tex cells in OC, which will contribute to the development of more precise and effective therapies.

## Data availability statement

The original contributions presented in the study are included in the article/**Supplementary Material**. Further inquiries can be directed to the corresponding author.

## Author contributions

KW participated in literature research and writing, other authors participated in data analysis, and BL was responsible for the overall project design and adjustment. All authors contributed to the article and approved the submitted version.
