# Supplementary material for: Ovarian cancer-associated immune exhaustion involves SPP1+ T cell and NKT cell, symbolizing more malignant progression
Source: Front Endocrinol (Lausanne). 2023 Apr 18;14:1168245. doi: 10.3389/fendo.2023.1168245 (PMC10151681; doi:10.3389/fendo.2023.1168245)
Supplement: Supplementary file 1 [file Image_1.pdf]

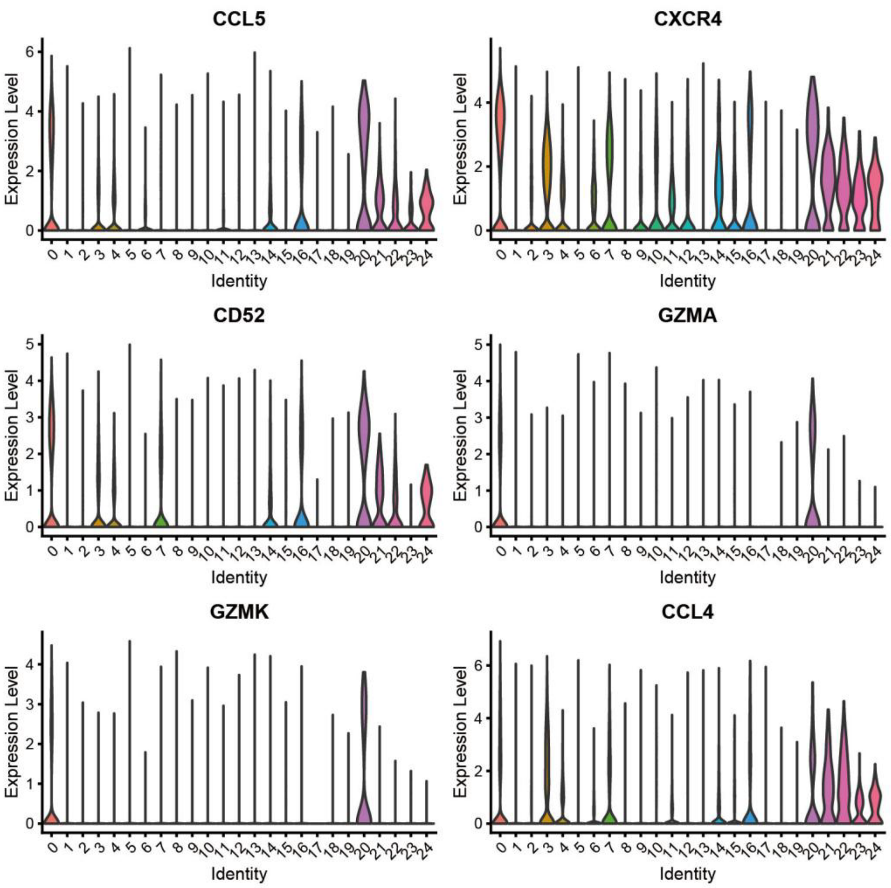
CCL5
CXCR4
CD52
GZMA
GZMK
CCL4
Expression Level
Identity

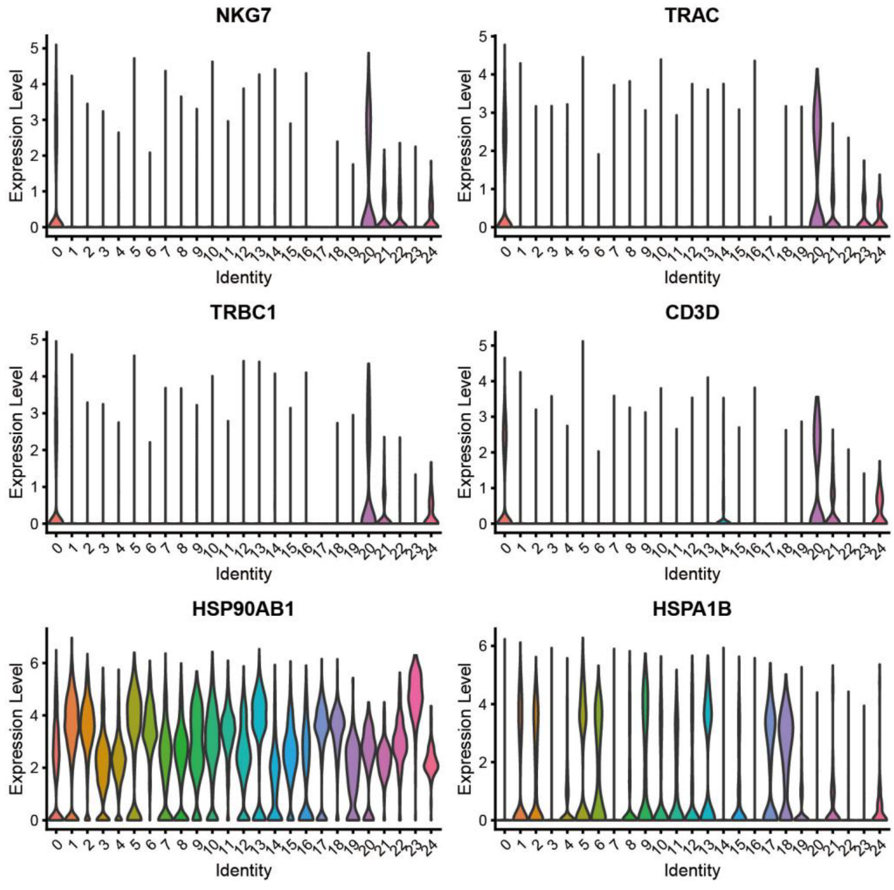
NKG7
TRAC
TRBC1
CD3D
HSP90AB1
HSPA1B
Expression Level
Identity

Supplementary figure 1. Violin plots show the expression of clustered signature genes in different groups.

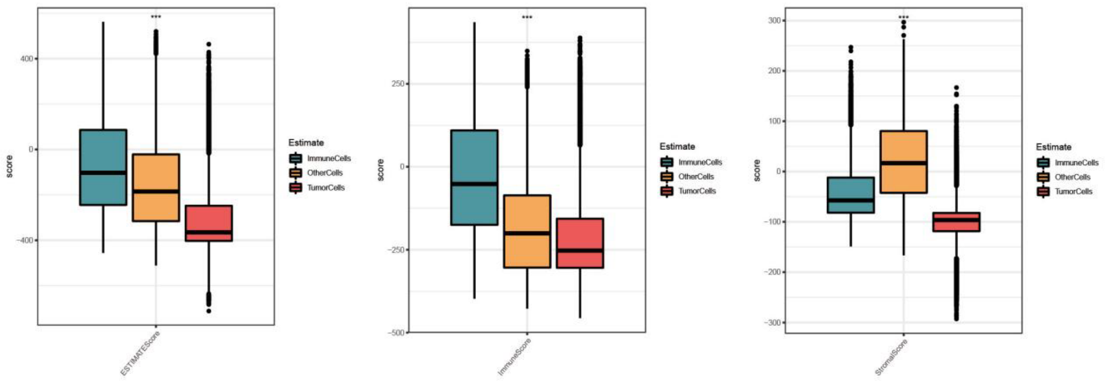

Supplementary figure 2. Box plots show the scores of ESTIMATE Score, Immune Score and Stromal Score for the three types of cells.

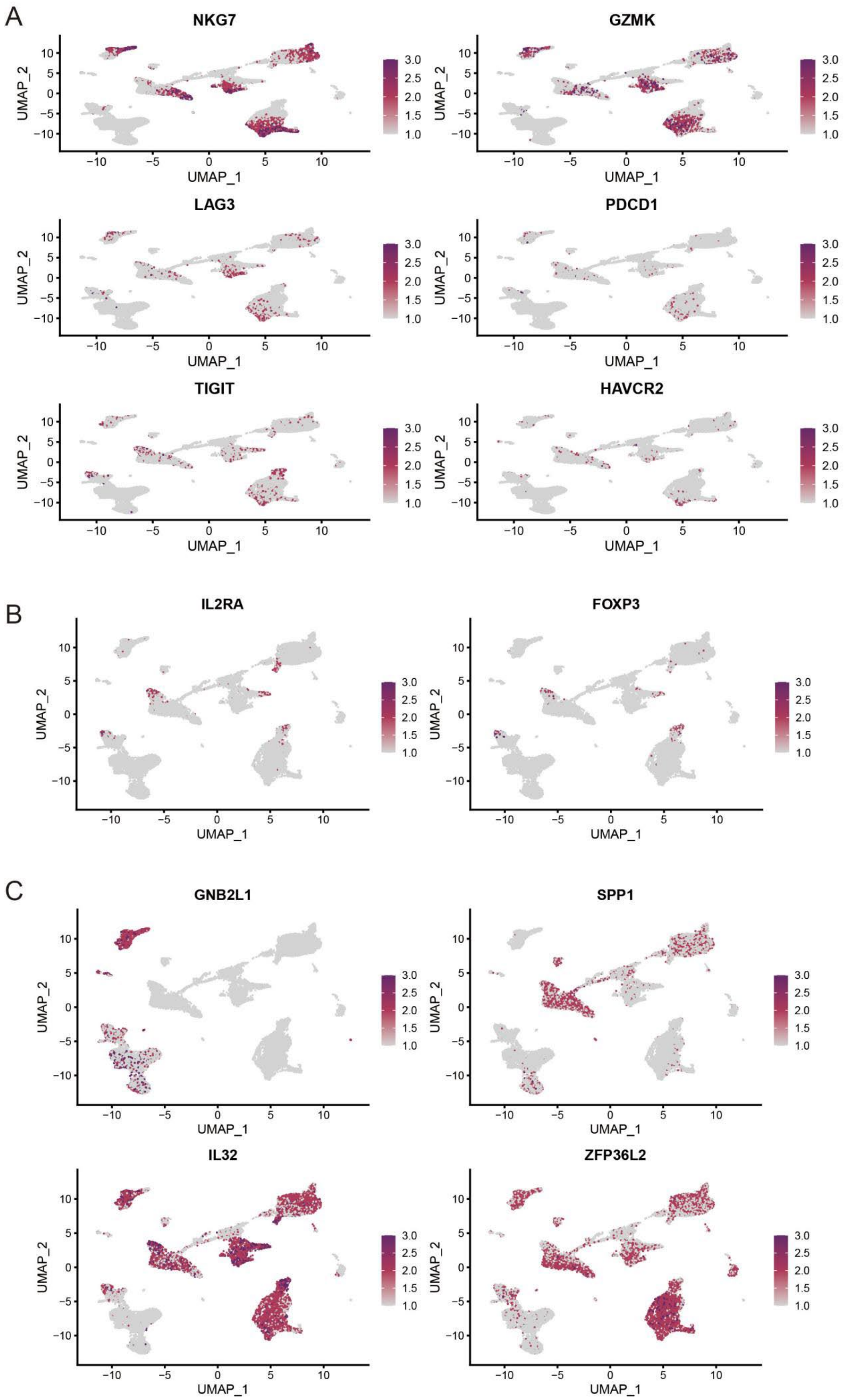
A
NKG7
GZMK
LAG3
PDCD1
TIGIT
HAVCR2
B
IL2RA
FOXP3
C
GNB2L1
SPP1
IL32
ZFP36L2
UMAP_1
UMAP_2
3.0
2.5
2.0
1.5
1.0
−10
−5
0
5
10

Supplementary figure 3. Umap plot showing the distribution of marker genes in signature cells. (A) Tex cells. (B) Treg cells. (C) Umap plot showing the distribution of highly expressed genes on Tex cells.

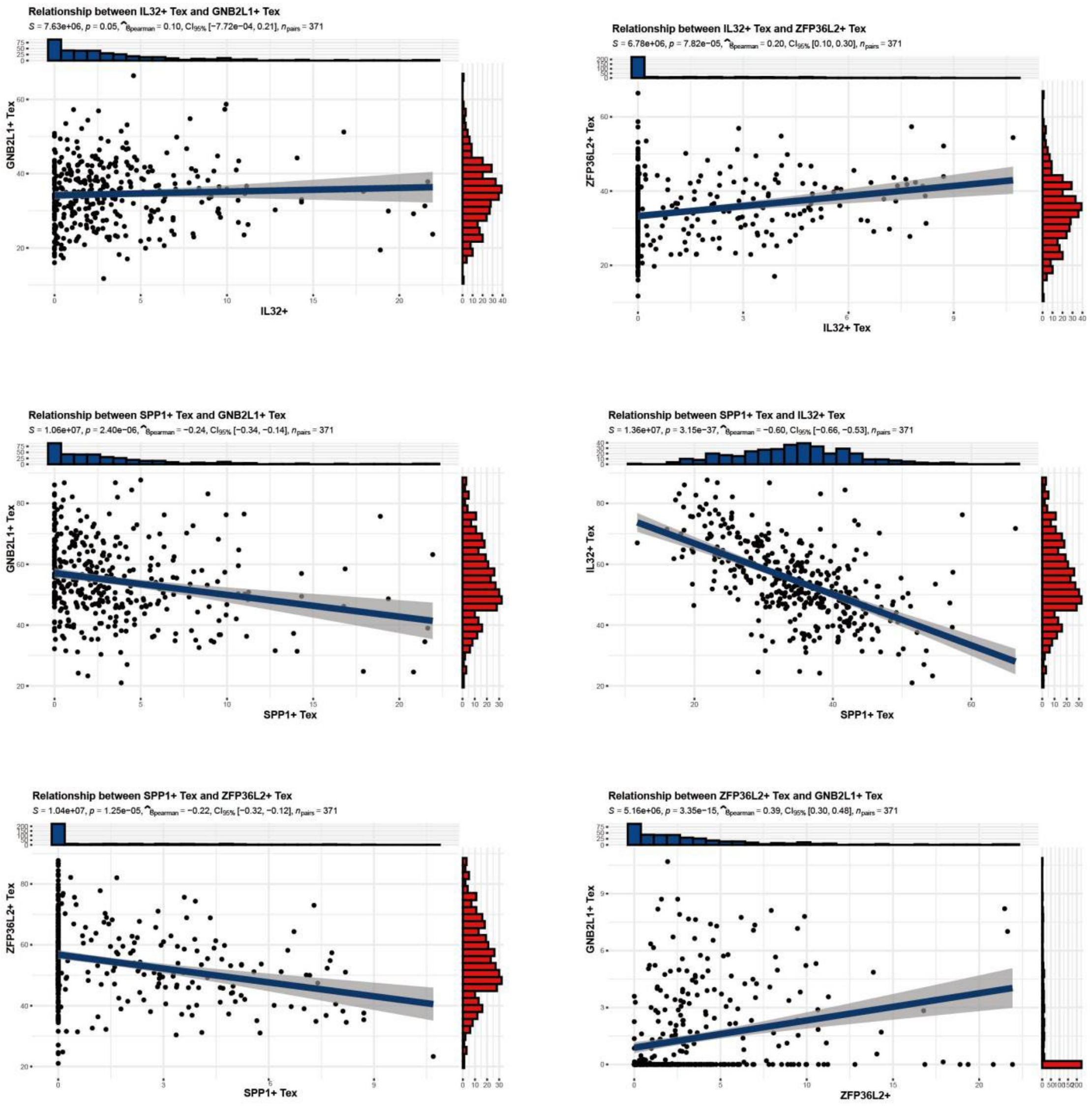

Supplementary figure 4. Correlation analysis between 4 Tex related cells.
